# Anti-IL17 antibody Secukinumab therapy is associated with ossification in giant cell tumor of bone: a case report of pathologic similarities and therapeutic potential similar to Denosumab

**DOI:** 10.1186/s12891-021-04182-z

**Published:** 2021-04-01

**Authors:** Andrew Chandler, Meredith K. Bartelstein, Tomohiro Fujiwara, Cristina R. Antonescu, John H. Healey, Max Vaynrub

**Affiliations:** 1 Department of Surgery, Orthopaedic Service, Memorial Sloan Kettering Cancer Center, 1275 York Ave., New York, NY 10065 USA; 2 Touro College of Osteopathic Medicine, New York, NY USA; 3 Department of Pathology, Memorial Sloan Kettering Cancer Center, New York, NY USA

**Keywords:** GCTB, Secukinumab, Denosumab, IL-17, EGFR, RANKL, Osteoprogeterin, Case report

## Abstract

**Background:**

Giant cell tumor of bone is a benign, locally aggressive neoplasm. Surgical resection is the preferred treatment method. However, for cases in which resection poses an increased risk to the patient, denosumab (anti-*RANKL* monoclonal antibody) is considered. Secukinumab is an anti-IL-17 antibody that is used in psoriatic arthritis to reduce bone resorption and articular damage.

**Case presentation:**

One case of giant cell tumor of bone (GCTB) in a patient treated with secukinumab for psoriatic arthritis demonstrated findings significant for intra-lesional calcifications. Histologic examination showed ossification, new bone formation, and remodeling. A paucity of osteoclast type giant cells was noted. Real-time quantitative polymerase-chain-reaction (qRT-PCR) analysis revealed decreased osteoclast function compared to treatment-naive GCTB.

**Conclusions:**

Secukinumab may play a role in bone remodeling for GCTB. Radiologists, surgeons, and pathologists should be aware of this interaction, which can cause lesional ossification. Further research is required to define the therapeutic potential of this drug for GCTB and osteolytic disease.

## Background

Giant cell tumor of bone (GCTB) is a benign, locally aggressive primary bone neoplasm [1]. Tumors typically arise in the epiphysis of the long bones, spine, and pelvis. On histology, many multinucleated giant cells are seen surrounded by neoplastic round or spindle-shaped mononuclear cells [1]. Recently, a histone 3.3 G34W mutation was described in GTCB and is used as a diagnostic marker [2]. The progression of GCTB is driven by the overproduction of receptor activator of nuclear factor kappa B (*NF-KB*) ligand (*RANKL*), thus driving osteoclastogenesis and bone resorption [3]. Surgical resection of GCTB is the preferred treatment method, but for cases in which resection poses an increased risk to the patient, denosumab (anti-*RANKL* monoclonal antibody) is considered [1]. Treatment with denosumab has been shown to decrease tumor volume and eliminate multinucleated giant cells while increasing bone formation [4]. However, studies have shown this treatment method is associated with an increased rate of local recurrence and should be used selectively [5].

Interleukin-17 (IL-17) is a cytokine released by T-helper 17 (Th17) cells, which induces inflammation and plays a role in bone remodeling [6]. In patients with psoriatic arthritis (PsA), IL-17 mediates bone resorption by enhancing *RANKL* expression in osteoblasts, synovial cells, and mesenchymal stem cells [7]. Treatment with secukinumab (anti-IL-17 antibody) in patients with PsA reduces articular damage [8]. Similar outcomes have been reported in patients with plaque psoriasis and ankylosing spondylitis [9]. In GCTB, IL-17 increases the expression of metallopeptidase 9 (*MMP9*), *RANKL*, and cathepsin-K (*CathK*), leading to osteoclastogenesis and local bone destruction [10]. The mechanism of IL-17 mediated osteoclastogenesis in GCTB may occur through activation of epidermal growth factor receptor. In triple-negative breast cancers (TNBCs), IL-17 maintains activation of EGFR, thus promoting tumor growth and evasion of anti-EGFR therapies [11]. EGFR promotes the progression of GCTB through osteoblast stromal proliferation and osteoclastogenesis [12]. We report a unique case of GCTB showing atypical histologic features in a patient treated with secukinumab for PsA. Pathological findings identified gross and microscopic ossification reminiscent of tumors treated with denosumab. The mechanism for this ossification was explored, comparing osteoblastic and osteolytic molecular markers from denosumab-treated and treatment-naïve GCTB samples.

## Case presentation

**Fig. 1:**
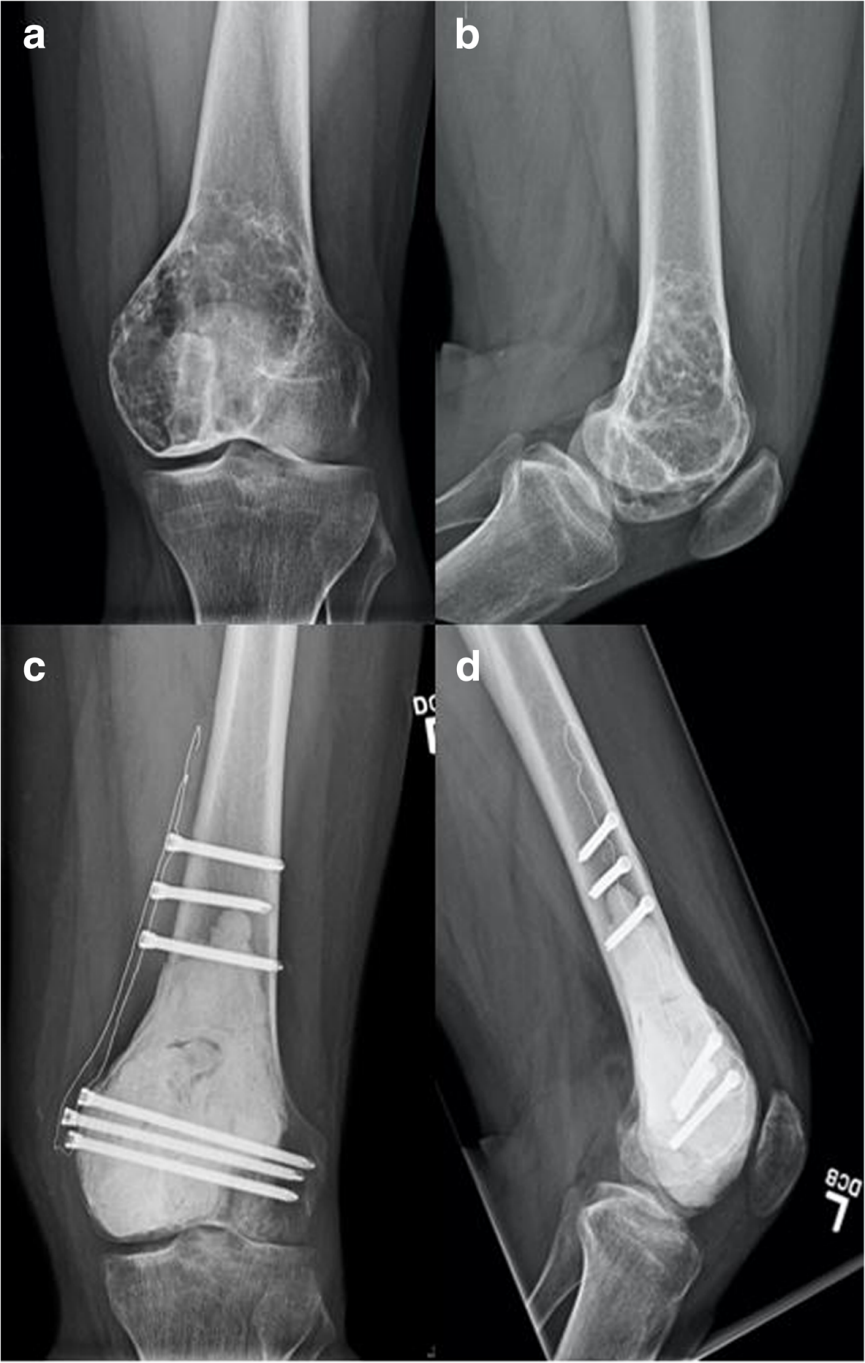
**a** Pre-surgical anteroposterior and **b** lateral radiographs of the distal femur demonstrating an eccentrically located mildly expansile lesion in the medial distal femoral metaphysis and epiphysis that is predominantly lytic with some internal and peripheral linear ossification components. **c** Post-surgical anteroposterior and **d** lateral radiographs representing intralesional curettage, adjuvant treatment with burr and cryoablation, and cementation with internal fixation using a carbon fiber locking plate

**Fig. 2:**
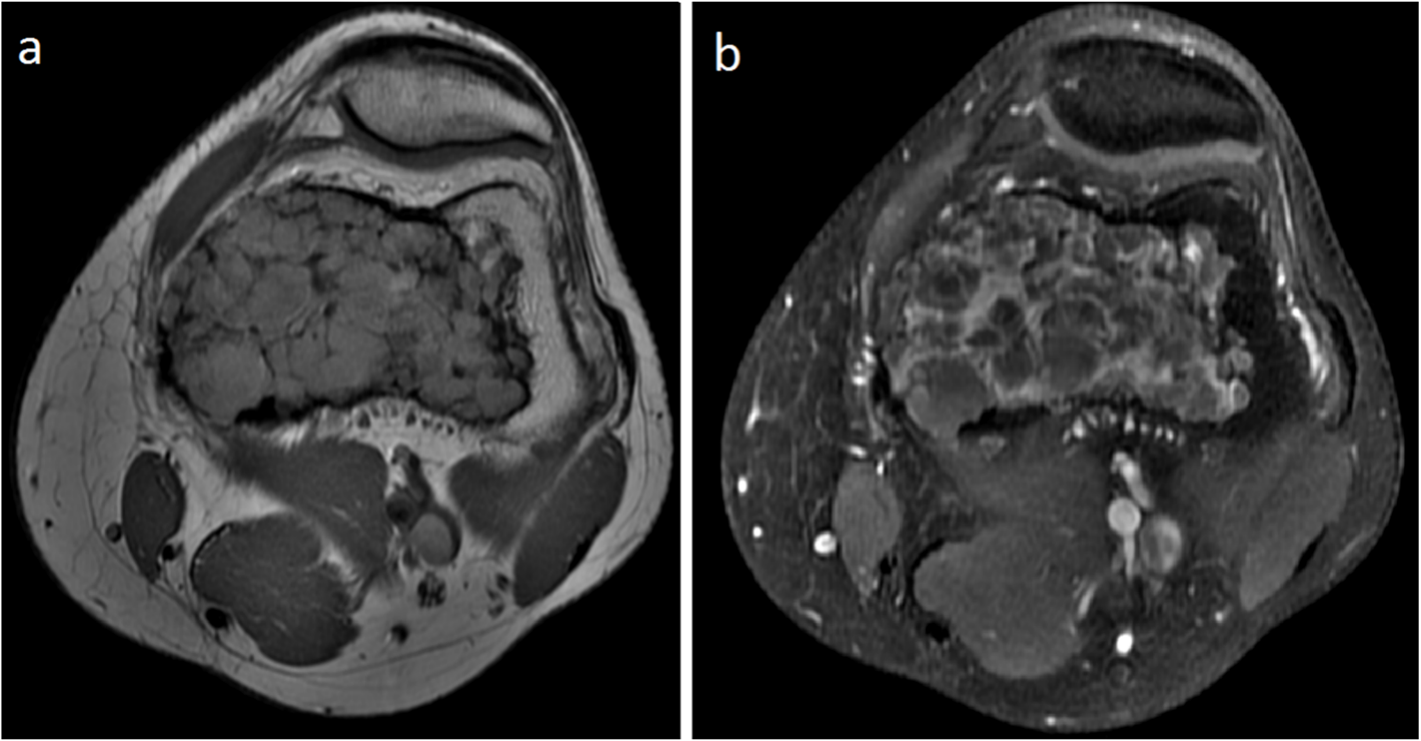
**a** Axial T1 pre- and **b** post-contrast images showing an expansile multicystic lesion in the distal femoral metaphysis

**Fig. 3:**
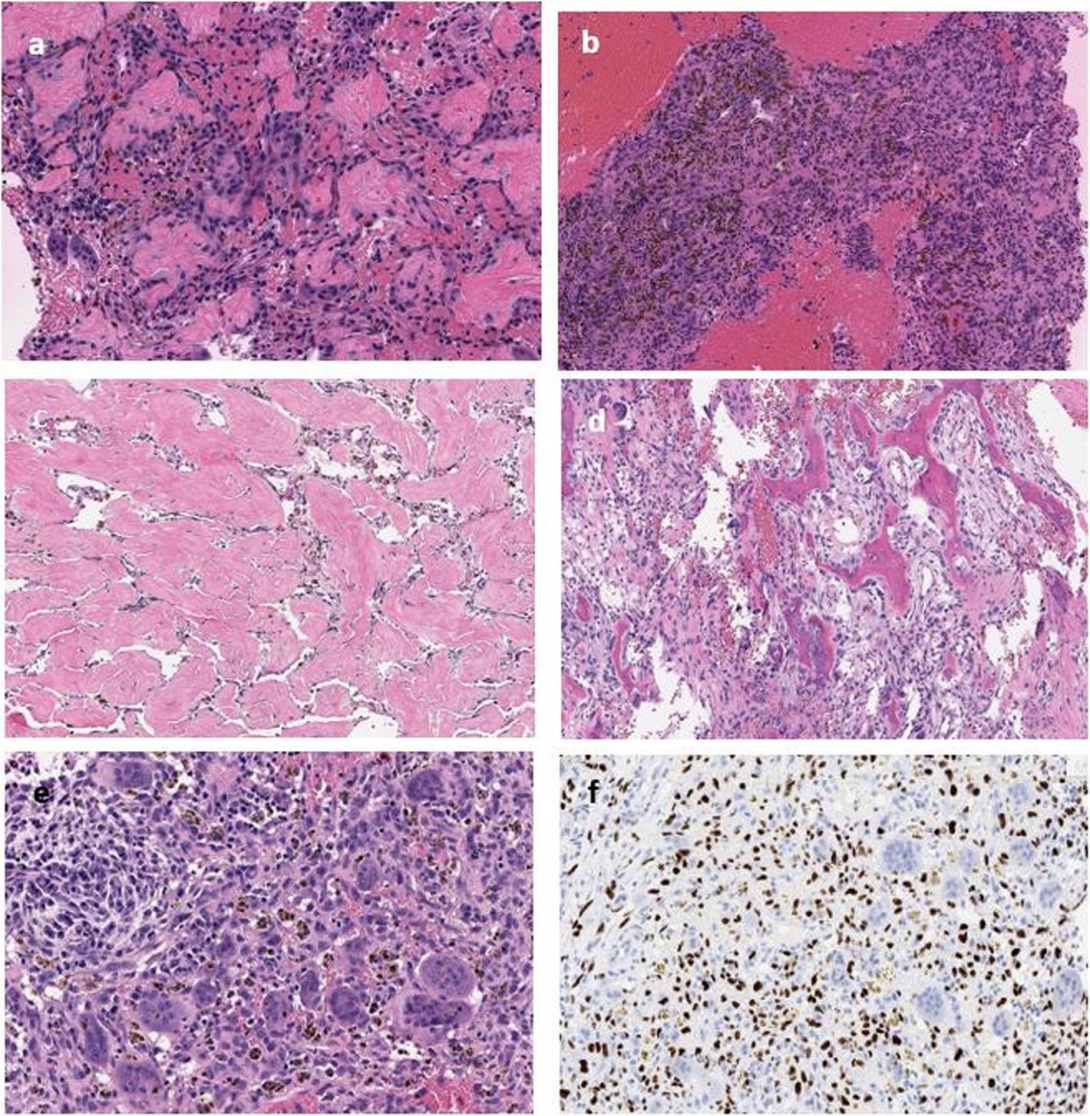
H&E histology. **a** Core needle biopsy specimen containing rare, multinucleated giant cells scattered within areas of new bone formation and osteoid matrix lined by osteoblastic riming. The very limited number of giant cells is not typical for a diagnosis of therapy-naïve GCTB. **b** Hemosiderin laden deposition; bloody spaces surrounded by cellular cyst wall. Findings are in keeping with a secondary aneurysmal bone cyst. **c** Material from the intralesional curetting shows confluent areas of osteoid matrix deposition, closely resembling the features seen in denosumab treated GCTB. **d** New woven bone formation lined by reactive osteoblastic cells, devoided by multinucleated osteoclasts. **e** Only one cellular focus showing a cluster of multinucleated giant cells were noted, admixed with mononuclear cells and hemosiderin deposition. **f** Immunohistochemical staining with H3.3G34W showed strong nuclear positivity in the neoplastic mononuclear cells, but negative in the reactive, surrounding multinucleated giant cells

A 32-year-old male presented with a six-month history of left knee pain and was found to have a lesion in the left distal femur. The patient provided informed consent for participation in this study under Memorial Sloan Kettering Cancer Center’s (MSK’s) protocol #06–107. The patient discussed in this report was informed data concerning the case would be submitted for publication and agreed. The patient had a seven-year history of PsA. This was initially treated for several years with adalimumab (Humira®) 20 mg subcutaneous every 14 days and intermittent methotrexate 15 mg orally per week. Due to persistent symptoms of PsA, he transitioned to secukinumab (Cosentyx®) 150 mg monthly prior to presentation. This was taken for a total of 2 months, and it was discontinued 1 month prior to presentation due to the new finding of a bony lesion of uncertain diagnosis. New onset left knee pain that differed from his chronic symptoms prompted an X-ray to be obtained, which showed a lytic lesion in the metaphysis and epiphysis of the medial femoral condyle with intact cortices (Fig. 1). Magnetic resonance imaging demonstrated a lesion with nodular enhancement and multicystic changes, suggestive of GCTB with secondary aneurysmal bone cyst (Fig. 2). A core needle biopsy confirmed the diagnosis of conventional GCTB (Fig. 3). Staging workup was negative for pulmonary disease. The patient underwent intralesional curettage, adjuvant treatment with high-speed burr and cryoablation, and cementation with internal fixation (Fig. 1).

On intraoperative gross examination, the solid, calcified lesional matrix was admixed with multiple thin-walled cystic spaces extending to the normal surrounding bone. Pathology examination identified multiple areas of reactive new bone formation (Fig. 3). Findings were not consistent with conventional GCTB that typically contains numerous osteoclast-like multinucleated giant cells. Rather, the presence of increased ossification and remodeling, with a decreased number of giant cells recapitulated the typical appearance seen in GCTB s/p denosumab therapy [13]. Confirmation of GCTB diagnosis was made by immunohistochemistry, showing strong nuclear positivity with H3.3G34W antibody in the mononuclear cells (Fig. 3). These findings prompted further investigation of what effect, if any, secukinumab had on the tumor.

To investigate this question, the secukinumab-treated tumor was compared microscopically and at gene expression level with two other conventional GCTB lesions: one treatment-naïve and one treated with five standard doses of denosumab (120 mg/dose). In both cases, the diagnosis was confirmed with H3.3G34W immunoreactivity.

In both the secukinumab and denosumab treated tumors, new bone formation and a limited number of multinucleated giant cells were seen on routine stains. In the case without biologic treatment, multinucleated giant cells were abundant, and there was no new bone formation.

**Fig. 4:**
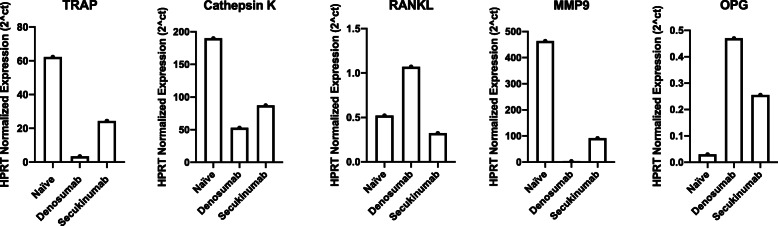
Comparative gene expression of three cases of GCTB with different treatment modalities. *TRAP*, *Cathepsin K*, *RANKL*, and *MMP9* represent osteoclast markers and function. Osteoprotegerin is a marker for osteoblast/bone formation

Gene expression of all three tumors was compared to better understand the landscape of tumor growth. Routine real-time quantitative polymerase-chain-reaction (qRT-PCR) analysis was used to quantitate mRNA expression in each tumor and was normalized to the housekeeping gene *HPRT* (Table 1). Four genes, *TRAP*, *CathK*, *RANKL*, and matrix *MMP9*, were measured to assess osteoclast function. In both secukinumab and denosumab treated tumors, *TRAP*, CathK, and *MMP9* expression was suppressed compared to the treatment-naïve tumor (Fig. 4). The effect was different for *RANKL*. This was likely due to the lack of direct negative feedback in the presence of denosumab. Osteoprotegerin (OPG) was used to assess osteoblast/bone formation function. Similarly, both immunologically treated tumors demonstrated increased OPG expression compared to the treatment-naïve tumor.

**Table 1:** Primers used in qRT-PCR^a^ analysis to assess osteoclast and osteoblast function

Gene Name	Forward Primer	Reverse Primer
*HPRT*	TGAGGATTTGGAAAGGGTG	GAGGGCTACAATGTGATGG
*TRAP*	GACCACCTTGGCAATGTCTCTG	TGGCTGAGGAAGTCATCTGAGTTG
*Cathepsin K*	TGAGGCTTCTCTTGGTGTCCATAC	AAAGGGTGTCATTACTGCGGG
*RANKL*	GAGAAAGCGATGGTGGATGG	CTTGCTCCTCTTGGCCAGAT
*MMP9*	TGGGGGGCAACTCGGC	GGAATGATCTAAGCCCAG
*OPG*	AGAAAGGAAATGCAACACACGAC	CCTGAAGAATGCCTCCTCACAC

^a^ *qRT-PCR* reverse transcription-quantitative polymerase-chain-reaction

At one-year post-operation, the patient reported painless range of knee motion and a full return to all activities. Surveillance imaging showed no signs of recurrent GCTB. The patient continues to undergo surveillance physical exam and imaging at regular intervals to monitor for tumor recurrence.

## Discussion and conclusion

GCTB is a benign, but locally aggressive, primary bone neoplasm driven by the overproduction of *RANKL*, leading to osteoclastogenesis and bone resorption. We report a unique GCTB displaying atypical morphologic features in a patient on two-month treatment with secukinumab for PsA. Histologic analysis showed gross and microscopic characteristics reminiscent of GCTB treated with denosumab.

### Secukinumab promotes new bone growth in GCTB

IL-17 mediates bone resorption in inflammatory mediated joint diseases (e.g. PsA and ankylosing spondylitis) [7, 9]. In these conditions, secukinumab is recommended for use to reduce bone resorption and joint erosions [8]. In GCTB, IL-17 signaling increases the expression of *MMP9*, *CathK*, and *RANKL*, thus promoting osteoclastogenesis and local bone destruction [10]. A potential mechanism of IL-17 mediated osteoclastogenesis in GCTB may occur through activation of EGFR. In cultures of peripheral blood mononuclear cells, the addition of EGF with M-CSF increases the number of TRAP+ osteoclast-like giant cells [12]. Also, higher numbers of osteoclasts are reported in EGFR+ GCTB cases [12]. Inhibition of EGFR in GCTB, in-turn, elicits denosumab-like effects [14]. IL-17 may activate EGFR in GCTB similar to what is observed in TNBCs [11]. In these tumors, IL-17 enhances the sensitivity of EGFR to EGF through crosstalk of Src/PYK2, as well as phosphorylating EGFR and inducing nuclear translocation, an integral part in the signaling cascade [11]. We demonstrate an association between inhibition of IL-17 signaling and new bone growth with decreased expression of *MMP9*, *CathK*, and *RANKL* in a denosumab-like manner. These findings suggest secukinumab, an anti-IL-17 monoclonal antibody, likely affects bone turnover modulation in GCTB*.* Additionally, the ossification was observed after the patient had received only two doses of secukinumab, compared to the five doses of denosumab in the comparison tissue, raising the possibility of a shorter treatment duration to elicit an ossification response.

### Therapeutic advantage of Secukinumab in GCTB

A clinical benefit of denosumab exists in patients with advanced or unresectable GCTB [4]. In an open-label, parallel-group, phase 2 trial, 65 of 159 (41%) surgically unsalvageable patients showed an objective tumor response defined as partial or complete after treatment with denosumab [15]. Therefore, denosumab therapy in association with or instead of surgery is recommended for select cases. However, prolonged treatment with denosumab is associated with osteonecrosis of the jaw, peripheral neuropathy, skin rash, hypophosphatemia, and atypical femoral fracture [15]. Furthermore, the use of denosumab is contraindicated in pregnancy [16]. Yet, secukinumab has much milder adverse effects, including nasopharyngitis, headache, nausea, diarrhea, and pyrexia, and is not contraindicated for pregnant patients [17, 18]. Notably, secukinumab has not been associated with untoward bone effects, like osteonecrosis of the jaw or atypical fractures of the femur. A recent, comprehensive literature review of the clinical experience with secukinumab showed a consistent increase in spine and hip bone mineral density and no change in fracture incidence [19]. If secukinumab has a similar therapeutic advantage to that of denosumab, it may be a promising option for patients who experience adverse events or have contraindications to denosumab therapy.

The use of denosumab has been associated with an increased risk of GCTB recurrence after treatment. It is still unknown how denosumab increases recurrence, but it is thought that denosumab principally targets the reactive infiltrating giant cells rather than affecting the neoplastic spindle cells. By increasing ossification, tumor cells can be trapped and sequestered from curettage, contributing to recurrence [20]. Cells left behind may be the source of recurrence or malignant transformation. Whether secukinumab has a similar influence on recurrence risk remains unknown. The atypical findings of bone formation in the setting of inhibition of IL-17, a known modulator of osteoclastogenesis in GCTB, raises the possibility of a therapeutic benefit of secukinumab in GCTB. Due to the uncontrolled nature of this case report, we do not have access to radiographic or pathologic data from this patient’s tumor prior to treatment with secukinumab. Therefore, it is impossible to assign causality definitively or to rule out that his prior treatment with adalimumab was a contributing factor to the ossification in the tumor. Further studies are needed to investigate if there is a role for secukinumab as medical monotherapy or adjuvant therapy for select cases of GCTB.

## Data Availability

All data will be made available by the corresponding author by reasonable request.

## Abbreviations

*CathK*: Cathepsin K
IL-17: Interleukin − 17
GCTB: Giant cell tumor of bone
*MMP9*: Metallopeptidase 9
MSK: Memorial Sloan Kettering Cancer Center
*NFKB*: Nuclear factor kappa B
OPG: Osteoprotegerin
PsA: Psoriatic arthritis
qRT-PCR: Quantitative polymerase-chain reaction
Th17: T-helper 17
TNBCs: Triple negative breast cancers
